# Phycocyanin Exerts Anti-Proliferative Effects through Down-Regulating TIRAP/NF-κB Activity in Human Non-Small Cell Lung Cancer Cells

**DOI:** 10.3390/cells8060588

**Published:** 2019-06-14

**Authors:** Shuai Hao, Shuang Li, Jing Wang, Yan Yan, Xin Ai, Jiawen Zhang, Yuqing Ren, Tingting Wu, Liyun Liu, Chengtao Wang

**Affiliations:** 1 Beijing Advanced Innovation Center for Food Nutrition and Human Health, Beijing Engineering and Technology Research Center of Food Additives, Beijing Technology and Business University, Beijing 100048, China; haoshuai@btbu.edu.cn (S.H.); lishuangldw@163.com (S.L.); trotwj960@163.com (J.W.); 15128470659@163.com (Y.Y.); 18087740320@163.com (X.A.); zhangjiawen98@outlook.com (J.Z.); RYQ512@163.com (Y.R.); m18810529269@163.com (T.W.); 2 State Key Laboratory of Infection Disease Prevention and Control, National Institute for Communicable Disease Control and Prevention, Collaborative Innovation Center for Diagnosis and Treatment of Infectious Disease, Chinese Center for Disease Control and Prevention, Beijing 102206, China

**Keywords:** phycocyanin, non-small cell lung cancer, TIRAP, proliferation, NF-κB activity

## Abstract

Phycocyanin is a type of marine functional food additive, exerting a health care efficacy with no side effects. It has been shown that phycocyanin possesses anticancer function in non-small cell lung cancer (NSCLC) cells, but the underlying regulatory mechanism still remains unclear. Further investigation on the antineoplastic mechanism of phycocyanin would provide useful information on NSCLC treatment. In this study, we explored the in vitro function and mechanism of phycocyanin in three typical NSCLC cell lines, H1975, H1650, and LTEP-a2, for the first time. Phenotypic experiments showed that phycocyanin significantly induced the apoptosis as well as suppressed the growth of NSCLC cells. Transcriptome analysis suggested that toll/interleukin 1 receptor domain-containing adaptor protein (TIRAP) was significantly down-regulated by phycocyanin. Strikingly, similar to phycocyanin-treated assays, siRNA knockdown of TIRAP expression also resulted in the anti-proliferative phenomenon in NSCLC cells. In addition, the activity of NF-κB signaling was also suppressed after silencing TIRAP expression, revealing that phycocyanin exerted anti-proliferative function through down-regulating TIRAP/NF-κB activity in NSCLC cells. Collectively, this study has laid a theoretical basis on the treatment of NSCLC and the potential utilization of marine functional products.

## 1. Introduction

As the leading cause of cancer death worldwide, lung cancer continues to impose a major burden on healthcare systems and causes significant challenges for clinicians and patients [1]. Non-small cell lung cancer (NSCLC), a subtype of lung cancer, constitutes about 85–90% of lung cancer and is the most common cause of cancer death [2]. Most patients present with advanced disease at the time of diagnosis and have a very poor prognosis, with the vast majority surviving less than five years [3]. Although some new approaches such as targeted and immunotherapy have been introduced in NSCLC treatment [1], there still remains a major need for therapy that significantly extends patients survival without compromising quality of life.

Nowadays, extensive studies have revealed that many marine natural products derived from *Spirulina* and *Cyanobacteria* exert multiple potent biological functions, with less or no toxic side effects [4,5], which have become one of the most important resources of novel lead compounds for critical diseases [6]. Phycocyanin, one of the phycobiliproteins derived from *Spirulina*, can be used as an effective ingredient of food additives, functional healthcare food, cosmetics, and pharmaceuticals [7,8]. A wide range of investigations have suggested that phycocyanin exerts multiple biological activities such as antioxidant [9], anti-cancer [10], anti-inflammation [11], immunomodulatory [12], neuroprotective effects [13], and so on. Particularly, more studies have shown that phycocyanin exerts remarkable antineoplastic effects in various cancer cell types including liver cancer [14], breast cancer [15], ovarian cancer [16], colon cancer [17], and malignant melanoma [10]. As a type of important natural functional food additive, phycocyanin possesses great application value in the potential treatment of human cancers.

Besides the above mentioned tumors, phycocyanin also has great potential of NSCLC treatment. Baudelet et al. discovered that the glaucophyte *Cyanophora paradoxa* pigments exerted antiproliferative effects on multiple cancer cells including NSCLC A549 cells [18]. Li et al. investigated the synergistic regulatory effects of all-trans retinoic acid and phycocyanin. They found that all-trans retinoic acid could promote the anti-growth activity of phycocyanin on A549 cells [19,20]. In addition, Bingula et al. reported the anti-proliferative effects of phycocyanin and betaine on A549 cells [21]. It is worth noting that the above-mentioned studies merely investigated the biological functions of phycocyanin in a single cell line; the underlying regulatory mechanism of phycocyanin in NSCLC still remains unclear. Further exploration of its regulation approach would provide useful information on the potential treatment of NSCLC. In the present work, we, for the first time, systematically investigated the antineoplastic mechanism of phycocyanin in three typical NSCLC cells (H1975, H1650, and LTEP-a2 cells), which was expected to lay a theoretical foundation for the future treatment of NSCLC and the utilization of phycocyanin.

## 2. Materials and Methods

### 2.1. Cell line and Culture Condition

Human NSCLC cell lines H1975, H1650, and LTEP-a2 were purchased from American Type Cell Collection (ATCC, Manassas, VA, USA). Cells were cultured in RPMI-1640 media (Invitrogen, Carlsbad, CA, USA) containing 10% fetal bovine serum (Hyclone, Logan, UT, USA) at 37 °C in a humidified atmosphere with 5% CO_2_.

### 2.2. siRNA Transfection Assay

A siRNA transfection assay was performed as described in our former study [22]. Briefly, cells were seeded into 6-well plates, with an appropriate density beforehand, and transfected into 80 nM of a siRNA (GenePharma, Shanghai, China) for each well using DhamaFECT 1 reagent according to the manufacturer’s instructions (Dharmacon, Lafayette, CO, USA). Negative siRNA was used as the negative control. The cells were exposed to siRNA and the negative control for 12 h, followed by replacing media and proceeding with subsequent experiments. The sequence of the TIRAP siRNA was as follows: sense 5′-GGCAGACCCUGCUGAAGAATT-3′; anti-sense 5′-UUCUUCAGCAGGGUCUGCCTT-3′. The sequence of Neg. siRNA was as follows: sense 5′-GCGACGAUCUGCCUAAGAU-3′; anti-sense 5′-AUCUUAGGCAGAUCGUCGC-3′.

### 2.3. Cell Survival Rate Assay

A cell survival rate assay was detected by the 3-(4,5-Dimethylthiazol-2-yl)-2,5-diphenyltetrazolium bromide (MTT) method as described in our former study [10]. Briefly, cells were seeded at a density of 5000 cells in 100 µL of medium per well into 96-well plates. After overnight incubation, phycocyanin with different concentrations (0, 2, 4, 6, and 8 µM) was added into each well. The control cells (0 µM) were treated with equivalent phosphate buffer solution (PBS) as phycocyanin treatment cells. Four replicates were performed for each condition. After incubation for 24 h, the cultured medium was supplemented with 1 mg/mL MTT for 4 h at 37 °C, followed by media removal and dimethylsulfoxide (DMSO) addition. The absorbance was measured at 450 nm and 630 nm.

### 2.4. Cell Proliferation Assay

A cell proliferation assay was detected by the MTT method. Briefly, after incubation with phycocyanin for 24 h, cells were seeded at an appropriate density into 96-well plates the day before detection. Then, the treated cells were incubated with MTT for 4 h, followed by Sodium Dodecyl Sulfonate-HCl (SDS-HCl) solution addition on each day. The absorbance was detected at 570 nm and 630 nm. The proliferation assay lasted for 5 days. The control cells (non-phycocyanin treated) were treated with equivalent PBS. For the proliferation analysis of cells transfected with siRNA, cells were exposed to siRNA and the negative control for 12 h, followed by dissociation with trypsin and transferal into 96-well plates. Similar process was performed as described above. Three independent experiments were carried out.

### 2.5. Cell Colony Formation Assay

A cell colony formation assay was performed as described in Qian’s work [23]. Briefly, cells were seeded at about 300 cells per well in 6-well plates. After 12 h incubation, cells were treated for another 24 h with appropriate amounts of phycocyanin, followed by continuous incubation in fresh medium at 37 °C in a cell incubator. After 12–15 days, cells were washed with PBS twice, fixed with methanol for 15 min, and stained with 0.5% crystal violet for 15 min at room temperature. The number of cell colonies was counted for analysis. For the colony formation analysis of cells transfected with siRNA, cells were exposed to siRNA and the negative control for 12 h, followed by dissociation with trypsin and transferal into 6-well plates. Similar process was performed as described above. Three independent experiments were carried out.

### 2.6. Cell Cycle Assay

After being treated with phycocyanin for 48 h, cells were harvested and fixed in 1 mL 70% cold ethanol in tubes and incubated at 4 °C for at least 72 h. Cells were centrifugated at 1500 rpm for 5 min, and the cell pellets were resuspended in 500 µL of propidium iodide (PI)/RNase staining buffer, incubated on ice for 30 min, followed by washing twice with cold PBS. Cell cycle distribution was measured using FACSCalibur (Becton Dickinson, Franklin Lakes, NJ, USA). For the cell cycle analysis of cells transfected with siRNA, cells were exposed to siRNA and the negative control for 12 h, followed by culturing with complete RPMI-1640 media for 36 h before collecting. Cell collecting and fixing processes were performed as described above. Three independent experiments were carried out.

### 2.7. Cell Apoptosis Assay

After being treated with phycocyanin for 48 h, cells were harvested and washed twice with cold PBS and then resuspended in 500 µL binding buffer. Then, cells were stained in 5 µL Annexin V-FITC/PI according to the manufacturer’s protocol (Roche, Mannheim, Germany). Stained cells were analyzed by FACSCalibur (Becton Dicknson). For the cell apoptosis analysis of cells transfected with siRNA, cells were exposed to siRNA and the negative control for 12 h, followed by culturing with complete RPMI-1640 media for 36 h before collecting. Cell collecting and fixing processes were performed as described above. Three independent experiments were carried out.

### 2.8. RNA-Seq Analysis

The LTEP-a2 cell line was selected for RNA-seq analysis. The transcriptome sequencing was performed using the high-throughput sequencing platform of Illumina HiSeq 4000 (Illumina, San Diego, CA, USA). Briefly, after treatment with phycocyanin, total RNA was extracted using Trizol reagent (Invitrogen). Five micrograms of total RNA was used for analysis. Base calling was adopted to convert original sequencing images to sequential data. The human genome sequence and gene annotations were obtained from the UCSC Genome Website (http://genome.ucsc.edu/). The differentially expressed genes (DEGs) between phycocyanin-treatment and control cells were identified based on fragments per kilobases per millionreads (FPKM) using RSEM 1.2.31 [24]. DESeq was used to determine the false discovery rate (FDR) threshold (adjust *p* value). If adjust *p* value was less than 0.05, it was considered to be a significantly different expression level.

### 2.9. Quantitative RT-PCR (qRT-PCR) Analysis

After being treated with phycocyanin for 48 h, cells were collected for RNA extraction. Total RNA was extracted using Trizol reagent (Invitrogen). For each detected gene, 2 µg of total RNA was reverse transcribed and quantified with a SYBR Green Real-Time PCR Master Mix Kit (TaKaRa, Dalian, China). Reduced glyceraldehyde-phosphate dehydrogenase (GAPDH) was used as an endogenous control. The primers were as follows: TIRAP forward 5′-CAGGACAGCCCACTACCCCC-3′, reverse 5′-TTTGACTTGACGAAAGCCAC-3′; GAPDH forward 5′-ATCCCATCACCATCTTCCAG-3′, reverse 5′-CCATCACGCCACAGTTTCCC-3′. The relative expression of each gene was then calculated and normalized. For siRNA transfection, cells were exposed to siRNA and the negative control for 12 h, followed by culturing with complete RPMI-1640 media for 36 h before collecting. Cell collecting and RNA extraction processes were performed as described above. Each assay was performed in quadruplicate. Three independent experiments were carried out.

### 2.10. Western Blot Analysis

After being treated with phycocyanin for 72 h, cells were collected for protein extraction. Proteins were extracted using RIPA buffer with protease inhibitors (Roche) and quantified using the Bradford reagent (Biomed, Beijing, China). Equivalent amounts of protein were subjected to 12% SDS-PAGE separation and then electro-transferred to polyvinylidene difluoride (PVDF) membranes (Millipore, Germany). After blocking, the membranes were incubated with anti-TIRAP rabbit antibody (Catalog No. YT4667), anti-phospho-IKKα/β rabbit antibody (Catalog No. 2697), anti-phospho-p65 rabbit antibody (Catalog No. 3033), anti-phospho-IκB-α rabbit antibody (Catalog No. 2859), and anti-β-actin mouse antibody (Catalog No. 3700) at 4 °C overnight, followed by incubation with horseradish peroxidase-conjugated anti-rabbit IgG (Catalog No. 7074) or anti-mouse IgG (Catalog No. 7076) secondary antibodies. The anti-TIRAP antibody was purchased from ImmunoWay (ImmunoWay Biotechnology Company, Plano, TX, USA), and other antibodies were purchased from Cell Signaling Technology (Cell Signaling Technology, Danvers, MA, USA). Signals were detected using enhanced chemiluminescence (Millipore). For siRNA transfection, cells were exposed to siRNA and the negative control for 12 h, followed by culturing with complete RPMI-1640 media for 48 h before collecting. Cell collecting and protein extraction processes were performed as described above.

### 2.11. Statistical Analysis

The statistical analysis was performed using SPSS software and labeled by an asterisk (*, *p* < 0.05; **, *p* < 0.01). The experimental data are shown as means ± standard deviation (SD).

## 3. Results

### 3.1. Phycocyanin Suppressed the Growth and Viability of Non-Small Cell Lung Cancer Cells

**Figure 1:**
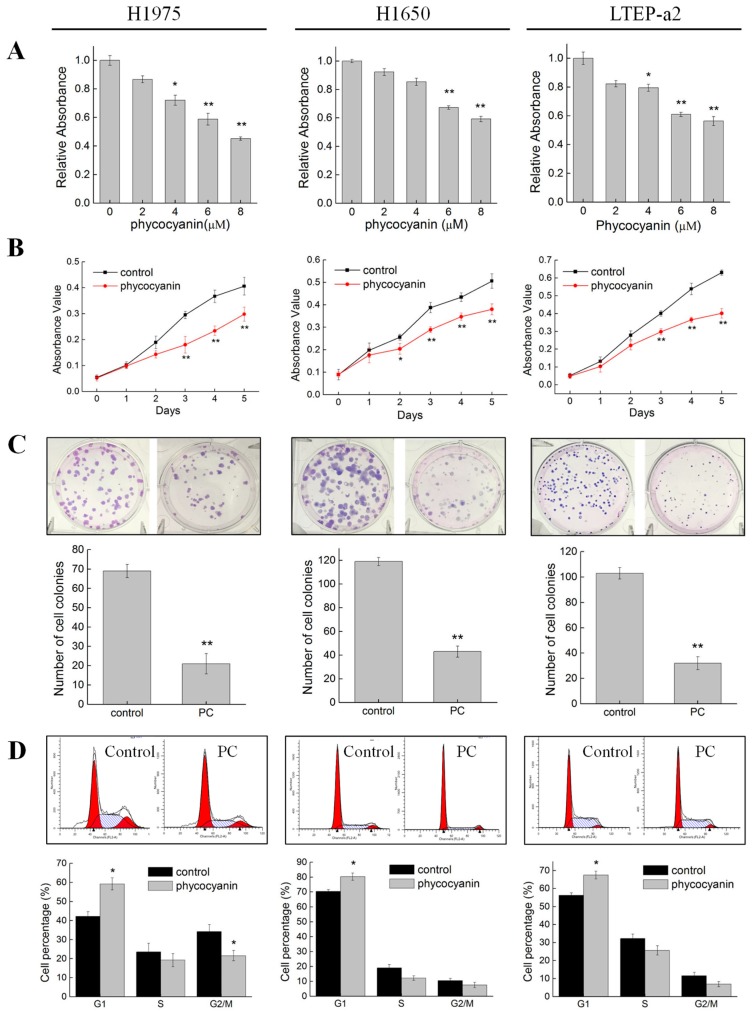
Phycocyanin suppressed the growth and viability of non-small cell lung cancer cells. (**A**) Survival rate assay of H1975, H1650, and LTEP-a2 cells after different concentrations of phycocyanin (0, 2, 4, 6, and 8 µM) treatment for 24 h. (**B**) Cell proliferation assay of H1975, H1650, and LTEP-a2 cells after 6 µM phycocyanin treatment for 24 h. The proliferation experiment lasted for five days. (**C**) Cell colony formation assay of H1975, H1650, and LTEP-a2 cells after 6 µM phycocyanin treatment for 24 h, followed by continuous incubation in fresh media. The cell colony formation experiment lasted for 12–15 days. (**D**) Cell cycle analysis of H1975, H1650, and LTEP-a2 cells after 6 µM phycocyanin treatment for 48 h. PC, phycocyanin treated group; control, non-phycocyanin treated group. Bars represent mean ± SD. *, *p* < 0.05; **, *p* < 0.01.

Phycocyanin was reported as an effective anti-cancer food additive as it could inhibit the growth of different cancer cells [14,15,16,17]. In the present study, to address the relationship between phycocyanin and its effects on multiple NSCLC cell lines, we first investigated the phenotypic experiments on H1975, H1650, and LTEP-a2 cells. As shown in Figure 1A, compared with control cells, incubation with phycocyanin dose-dependently (0, 2, 4, 6, and 8 µM) reduced the survival rate of H1975, H1650, and LTEP-a2 cells. The viability of the three NSCLC cells was significantly inhibited when phycocyanin concentration was 6 and 8 µM. In this case, we selected 6 µM as the treatment dose in the following experiments. Cell proliferation results showed that phycocyanin could significantly suppress the growth of NSCLC cells from the second (H1650 cell line) or the third (H1975 and LTEP-a2 cell lines) day (Figure 1B). In addition, to investigate the effects of phycocyanin on the unanchored growth ability of NSCLC cells, a clonogenic assay was performed. Figure 1C indicates that compared to control groups, phycocyanin-treated cells displayed significant reduction in colony formation, revealing the potent inhibition of cell growth and reproductive integrity. To further elucidate the mechanism of growth inhibition on NSCLC cells, the effects of phycocyanin on cell cycle progression were analyzed in H1975, H1650, and LTEP-a2 cells. Results showed that phycocyanin could cause significant changes in cell cycle distribution of NSCLC cells (Figure 1D). As compared to the control groups (42.23% ± 2.34%, 70.32% ± 1.23%, and 56.23% ± 1.43% in H1970, H1650, and LTEP-a2 cells, respectively), after treatment with phycocyanin, the proportion of G1 phase cells reached 59.21% ± 3.12%, 80.32% ± 2.45%, and 67.46% ± 2.14% in H1975, H1650, and LTEP-a2 cells, respectively, suggesting that phycocyanin induced G1 phase arrest in these three cells. Particularly, this was consistent with Madamwar’s work in A549 cells [25]. Taken together, these results strongly suggested that phycocyanin exerted significant anti-proliferative effects on NSCLC H1975, H1650, and LTEP-a2 cell lines.

### 3.2. Phycocyanin Induced Apoptosis of Non-Small Cell Lung Cancer Cells

**Figure 2:**
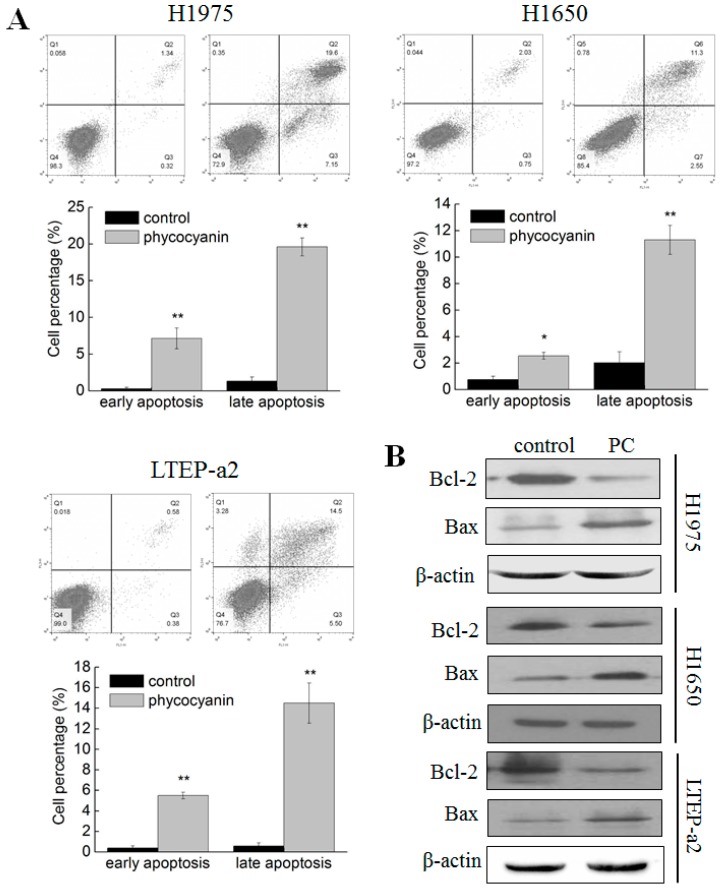
Phycocyanin induced apoptosis of non-small cell lung cancer cells. (**A**) Cell apoptosis analysis of H1975, H1650, and LTEP-a2 cells after treatment with 6 µM phycocyanin for 48 h. The proportion of early and late apoptotic cells are shown in the histogram. (**B**) Western blot analysis of the expressions of Bcl-2 and Bax in H1975, H1650, and LTEP-a2 cells after treatment with 6 µM phycocyanin for 72 h. PC, phycocyanin treatment groups. Bars represent mean ± SD. *, *p* < 0.05; **, *p* < 0.01.

As phycocyanin inhibited the growth of multiple NSCLC cells, we further studied its effects on apoptosis in H1975, H1650, and LTEP-a2 cells. As shown in Figure 2A, in comparison with control groups, phycocyanin exerted obvious apoptosis-promoting capacities in these three cell lines. The proportion of early and late apoptotic cells in H1975 (7.15% ± 1.42% and 19.6% ± 1.24%, respectively), H1650 (2.55% ± 0.25% and 11.3% ± 1.10%, respectively) and LTEP-a2 (5.50% ± 0.32% and 14.5% ± 1.96%, respectively) significantly increased after incubation with 6 µM phycocyanin. In addition, the expressions of two apoptotic markers (Bcl-2 and Bax) were tested using Western blot. As shown in Figure 2B, phycocyanin could reduce the expressions of oncogene Bcl-2 and increase the expressions of tumor suppressor gene Bax in H1975, H1650, and LTEP-a2 cells, which further supported the cell apoptosis results (Figure 2A). Taken together, the above results suggested that phycocyanin could induce apoptosis in multiple NSCLC cell lines.

### 3.3. Transcriptome Analysis Suggested TIRAP Was Down-Regulated by Phycocyanin in Non-Small Cell Lung Cancer Cells

**Figure 3:**
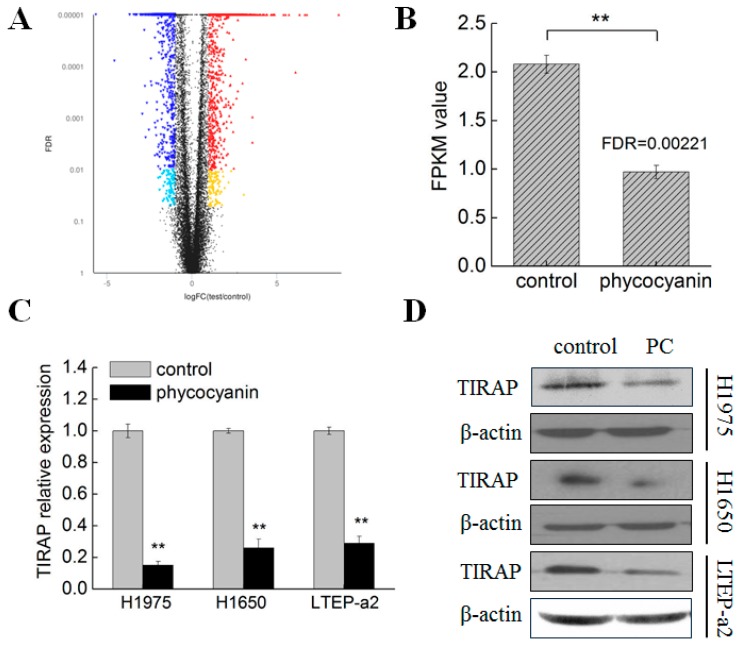
Phycocyanin reduced the expressions of TIRAP in non-small cell lung cancer cells. (**A**) RNA-seq analysis of differentially expressed genes between control and phycocyanin-treated LTEP-a2 cells. The horizontal axis indicates expression changes (log) of the genes and the vertical axis indicates the false discovery rate (FDR, adjust *p* value). Black dots are genes with no significant discrepancy. Dark and light blue dots are genes significantly down-regulated with FDR < 0.01 or 0.01 < FDR < 0.05, respectively. Red and yellow dots are genes significantly up-regulated with FDR < 0.01 or 0.01 < FDR < 0.05, respectively. (**B**) RNA-seq analysis of TIRAP expression in control and phycocyanin-treated LTEP-a2 cells. The duration of phycocyanin treatment was 48 h before cell collection and RNA extraction. (**C**) qRT-PCR analysis of TIRAP expressions in H1975, H1650, and LTEP-a2 cells after phycocyanin treatment. (**D**) Western blot analysis of TIRAP expressions in H1975, H1650, and LTEP-a2 cells after phycocyanin treatment for 72 h. PC, phycocyanin treatment groups; TIRAP, TIR domain-containing adaptor protein. Bars represent mean ± SD. *, *p* < 0.05; **, *p* < 0.01.

Phycocyanin was proven to exerted anti-proliferative and pro-apoptotic functions in NSCLC H1975, H1650, and LTEP-a2 cells. To gain a deeper insight into the anti-cancer mechanism of phycocyanin in NSCLC, we performed RNA-seq analysis in LTEP-a2 cells after 6 µM phycocyanin treatment. A rigorous comparison at adjust *p* < 0.05 and log_2_FC fold change of ≤−1 (down-regulation) or ≥1 (up-regulation) was made to identify the DEGs for different groups (Figure 3A). In total, gene expression analysis showed that 1640 genes were significantly differentially expressed, including 721 up-regulated and 919 down-regulated genes. Strikingly, toll/interleukin 1 receptor domain-containing adaptor protein (TIRAP) was discovered as a significant down-regulated differential protein (adjust *p* value = 0.00221) in phycocyanin-treated LTEP-a2 cells, as compared with control groups (Figure 3B). TIRAP is an important adaptor protein which belongs to the TLR/IL-1R superfamily, possessing a TIR domain in the cytoplasmic tail [26]. Several studies had indicated that TIRAP was involved in multiple biological processes including inflammation [27], cell growth [28], and apoptosis [29]. To further validate whether phycocyanin could decrease the expression of TIRAP in LTEP-a2 and other NSCLC cell lines, we performed qRT-PCR and Western analysis in phycocyanin-treated cells. As shown in Figure 3C,D, phycocyanin could significantly reduce the transcription and protein levels of TIRAP in LTEP-a2, H1975, and H1650 cells, suggesting that TIRAP might be involved in phycocyanin-mediated growth inhibition process in NSCLC cells. Besides TIRAP, we also validated the expressions of TRAF1, ICAM1, RELB, TNFAIP3, TLR4, MYD88, and IL1R1 in LTEP-a2 cells by qRT-PCR (Figure S1). These genes were related to NF-κB signaling and significantly regulated by phycocyanin in the transcriptome assay. As shown in Figure S1, the expressions of TRAF1, ICAM1, RELB, and TNFAIP3 significantly increased while the expressions of TLR4, MYD88, and IL1R1 decreased after phycocyanin addition. This was consistent with the RNA-seq results, which further supported our investigation.

### 3.4. Knockdown of TIRAP Expression Suppressed Proliferation of Non-Small Cell Lung Cancer Cells

**Figure 4:**
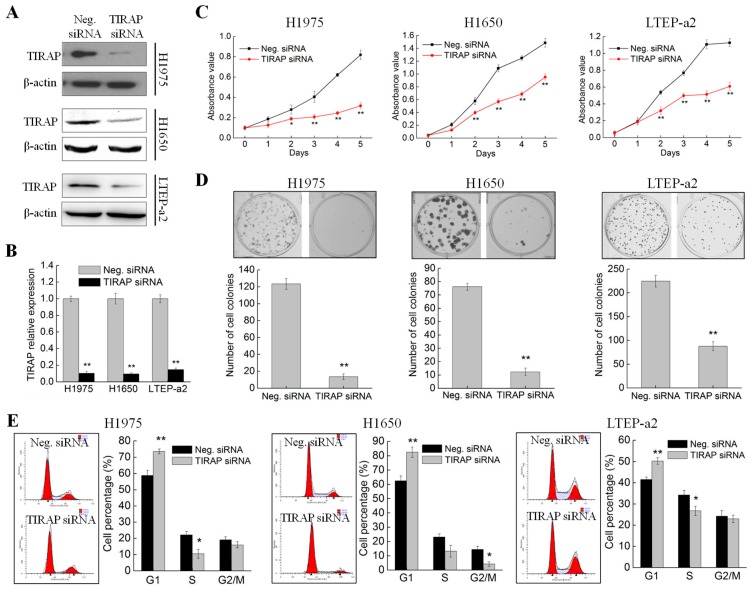
Knockdown of TIRAP expression suppressed the proliferation of non-small cell lung cancer cells. (**A**) Western blot analysis of TIRAP expressions in H1975, H1650, and LTEP-a2 cells after transfection with Neg. and TIRAP siRNA. All cells were exposed to Neg. and TIRAP siRNA for 12 h, followed by culturing with complete medium for 48 h before protein extraction and Western analysis. (**B**) qRT-PCR analysis of TIRAP expressions in H1975, H1650, and LTEP-a2 cells after transfection with Neg. and TIRAP siRNA. All cells were exposed to Neg. and TIRAP siRNA for 12 h, followed by culturing with complete medium for 36 h before RNA extraction and qRT-PCR analysis. (**C**) Cell proliferation assay of H1975, H1650, and LTEP-a2 cells after transfection with Neg. and TIRAP siRNA. All cells were exposed to Neg. and TIRAP siRNA for 12 h, followed by dissociation with trypsin and transferring into 96-well plates for proliferation analysis. (**D**) Cell colony formation assay of H1975, H1650, and LTEP-a2 cells after transfection with Neg. and TIRAP siRNA. All cells were exposed to Neg. and TIRAP siRNA for 12 h, followed by dissociation with trypsin and transferring into 6-well plates for colony formation analysis. (**E**) Cell cycle analysis of H1975, H1650, and LTEP-a2 cells after transfection with Neg. and TIRAP siRNA. All cells were exposed to Neg. and TIRAP siRNA for 12 h, followed by culturing with complete RPMI-1640 media for 36 h before cell cycle analysis. TIRAP, Toll/interleukin 1 receptor domain-containing adaptor protein. Neg. siRNA, negative control siRNA. Bars represent mean ± SD. *, *p* < 0.05; **, *p* < 0.01.

Phycocyanin was discovered to inhibit the expressions of TIRAP in NSCLC cells (Figure 3C,D). To further investigate whether phycocyanin exerted an anti-proliferative function through regulating TIRAP, we performed TIRAP knockdown experiments using specific siRNA. The transfection effect was validated by Western blot and qRT-PCR. As shown in Figure 4A,B, siRNA transfection significantly decreased the expressions of TIRAP in H1975, H1650, and LTEP-a2 cells, indicating that TIRAP was successfully silenced. Subsequently, cell proliferation, colony formation, and cell cycle assays were performed in these three cell lines. As shown in Figure 4C, siRNA knockdown of TIRAP could significantly restrain the proliferation of H1975, H1650, and LTEP-a2 cells from the second day. Figure 4D indicates that TIRAP knockdown also significantly inhibited the colony formation abilities of NSCLC cells. In addition, TIRAP knockdown caused changes in cell cycle distribution of NSCLC cells (Figure 4E). As compared with the control groups (58.76% ± 3.24%, 62.45% ± 3.42%, and 41.56% ± 1.14% in H1970, H1650, and LTEP-a2 cells, respectively), after transfection with TIRAP siRNA, the proportion of G1 phase cells reached 73.58% ± 1.54%, 82.56% ± 3.65%, and 50.27% ± 1.59% in H1975, H1650, and LTEP-a2 cells, respectively, suggesting that knockdown the expression of TIRAP led to G1 phase arrest in NSCLC cells. Taken together, the above results were highly in accord with the proliferation phenotype in phycocyanin-treated cells, indicating that phycocyanin could exert anti-proliferative function through down-regulating TIRAP in NSCLC cell lines.

### 3.5. Knockdown of TIRAP Expression Induced Apoptosis of Non-Small Cell Lung Cancer Cells

**Figure 5:**
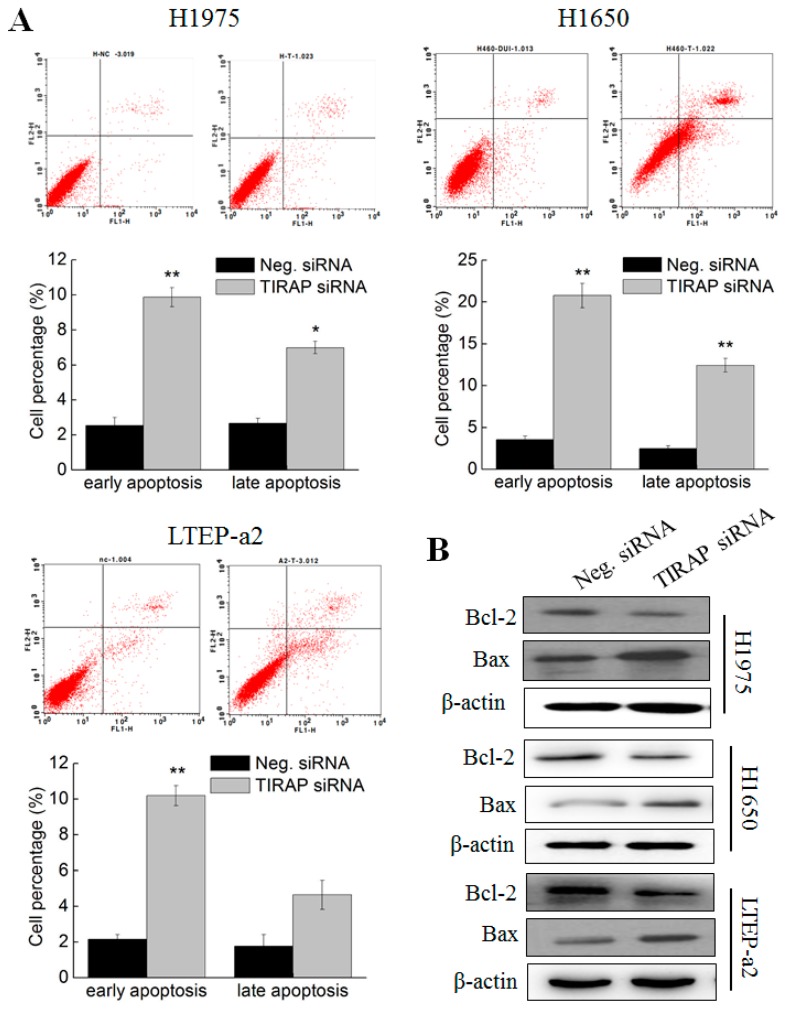
Knockdown of TIRAP expression induced apoptosis of non-small cell lung cancer cells. (**A**) Cell apoptosis analysis of H1975, H1650, and LTEP-a2 cells after transfection with Neg. and TIRAP siRNA. All cells were exposed to Neg. and TIRAP siRNA for 12 h, followed by culturing with complete RPMI-1640 media for 36 h before cell apoptosis analysis. The proportion of early and late apoptotic cells are shown in the histogram. (**B**) Western blot analysis of the expressions of Bcl-2 and Bax in H1975, H1650, and LTEP-a2 cells after transfected with Neg. and TIRAP siRNA. All cells were exposed to Neg. and TIRAP siRNA for 12 h, followed by culturing with complete medium for 48 h before protein extraction and Western analysis. TIRAP, Toll/interleukin 1 receptor domain-containing adaptor protein. Neg. siRNA, negative control siRNA. Bars represent mean ± SD. *, *p* < 0.05; **, *p* < 0.01.

The cell apoptosis experiments were performed in TIRAP siRNA transfected NSCLC cells. As shown in Figure 5A, siRNA knockdown of TIRAP expression significantly promoted the proportion of early apoptotic H1975 (9.87% ± 0.55%), H1650 (20.78% ± 1.45%), and LTEP-a2 (10.19% ± 0.57%) cells, as compared with Neg. siRNA transfection groups (2.54% ± 0.47%, 3.56% ± 0.43%, and 2.16% ± 0.26 in H1975, H1650, and LTEP-a2 cells, respectively), indicating that TIRAP participated in the apoptotic regulatory processes in multiple NSCLC cells. Particularly, silencing of TIRAP expression also caused significant increasing of late apoptosis cells in H1975 (6.99% ± 0.36%) and H1650 (12.44% ± 0.82%) cell lines. Likewise, TIRAP knockdown induced up-regulation of Bax and down-regulation of Bcl-2 expressions, respectively (Figure 5B). Therefore, combined with the results in Figure 2, these results suggested that TIRAP was involved in the phycocyanin-mediated apoptosis-inducing effects in H1975, H1650, and LTEP-a2 cells.

### 3.6. Phycocyanin Exerted Anti-Proliferative Effects through Down-Regulating TIRAP/NF-κB Activity in Non-Small Cell Lung Cancer Cells

**Figure 6:**
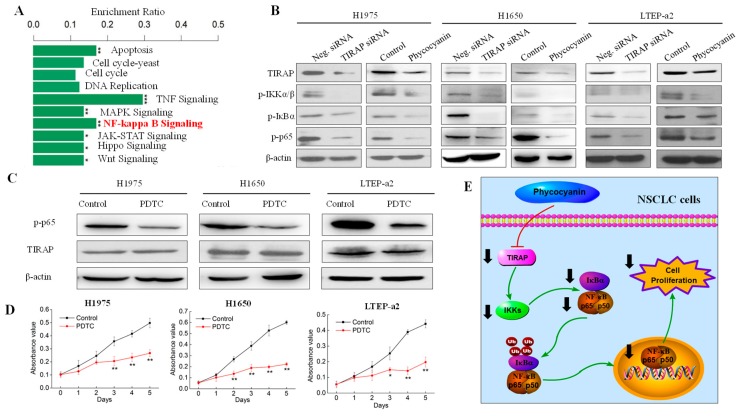
Phycocyanin exerted anti-proliferative effects through down-regulating TIRAP/NF-κB activity in non-small cell lung cancer cells. (**A**) RNA-seq analysis of the potential pathways involved in phycocyanin-mediated biological process in LTEP-a2 cells. *, *p* < 0.05, **, *p* < 0.01, ***, *p* < 0.001. (**B**) Western blot analysis of TIRAP and NF-κB pathway expressions in H1975, H1650, and LTEP-a2 cells after treatment with TIRAP siRNA and phycocyanin, respectively. For phycocyanin treatment, proteins were extracted after treatment with phycocyanin and control for 72 h. For siRNA transfection, cells were exposed to Neg. and TIRAP siRNA for 12 h, followed by culturing with complete medium for 48 h before protein extraction. (**C**) Western blot analysis of TIRAP and phosphorylated p65 expressions in H1975, H1650, and LTEP-a2 cells at 72 h after treatment with 10 µM PDTC for 24 h. (**D**) Cell proliferation analysis of H1975, H1650, and LTEP-a2 cells after treatment with 10 µM PDTC for 24 h. (**E**) Illustration of phycocyanin/TIRAP/NF-κB regulation process in NSCLC cells. TIRAP, Toll/interleukin 1 receptor domain-containing adaptor protein. Neg. siRNA, negative control siRNA. PDTC, pyrrolidine dithiocarbamate. Bars represent mean ± SD. *, *p* < 0.05; **, *p* < 0.01.

RNA-seq analysis indicated that NF-κB pathway activity was significantly decreased after phycocyanin treatment (Figure 6A). On the basis of the transcriptome results, the effects of phycocyanin and TIRAP on NF-κB signaling activity in NSCLC cells were examined. As shown in Figure 6B, besides TIRAP, phycocyanin decreased the phosphorylation levels of IKKα/β, IκBα, and p65 in H1975, H1650, and LTEP-a2 cells, which was in accord with Bingula’s study in A549 cells [21]. In addition, siRNA knockdown of TIRAP expression also inhibited the phosphorylation levels of these proteins, suggesting that TIRAP had regulatory effect on NF-κB pathway in NSCLC cells. To validate the above results, we also detected the total expressions and analyzed the phospho/total ratios of IKKα/β, IκBα, and p65 proteins (Figure S2). As expected, TIRAP siRNA and phycocyanin treatment could significantly decrease the phospho/total ratios of IKKα/β, IκBα and p65 in three cell lines, which further indicated a reduced activity of NF-κB signaling. To explore whether TIRAP exerted anti-proliferative effects on NSCLC cells through NF-κB pathway, we employed the pyrrolidine dithiocarbamate (PDTC) treatment experiment. PDTC is a NF-κB inhibitor, which could suppress the phosphorylation of IKKα/β and p65 [30]. As expected, blockage of NF-κB activity with PDTC significantly inhibited the proliferation of H1975, H1650, and LTEP-a2 cells (Figure 6C,D). Strikingly, PDTC treatment had no effect on TIRAP expressions, indicating that TIRAP is located on the upstream of NF-κB signaling (Figure 6C). Collectively, our work had revealed that phycocyanin could exerted anti-proliferative effects on multiple NSCLC cells through down-regulating TIRAP/NF-κB signaling (Figure 6E), which suggested a potential therapeutic approach for NSCLC, and provided a theoretical basis for the application of phycocyanin and functional foods.

## 4. Discussion

The antineoplastic activity of phycocyanin in NSCLC has been investigated for some time. Czerwonka et al. studied the anticancer potential of *Spirulina* extract (SE) against human NSCLC A549 cells [31]. They discovered that SE could cause G1 phase arrest and Bax to Bcl-2 ratio increasing in A549 cells, which was similar to our results (Figure 1 and Figure 2). Strikingly, *Spirulina* extract only contained about 12–19% phycocyanin [31], which from one side reflected the health-beneficial action of *Spirulina*. Likewise, Deniz and Thangam et al. also demonstrated the prevention capacities of phycocyanin against highly lethal lung cancer using the A549 cell model [32,33]. It is worth noting that Huang et al. investigated the antineoplastic activity of phycocyanin with a novel perspective by using a phycocyanin-based nanocarrier as a nanoplatform, which exerted highly efficient anticancer efficacy [34]. In addition, besides phycocyanin, phycoerythrin, which also derived from cyanobacterial, was proven to exerted anti-proliferative and pro-apoptotic effects on NSCLC A549 cells [35,36], indicating the potential pharmaceutical value of phycobiliproteins. Overall, as an important type of NSCLC cell line, A549 cells were widely used in multiple investigations on functional foods. Our study, for the first time, employed H1975, H1650, and LTEP-a2 NSCLC cell lines to explore the anticancer mechanisms of phycocyanin, which widely illustrated the antineoplastic capacity of phycocyanin in NSCLC cells.

NF-κB is a family of five master transcription factors, which can form various heterodimers or homodimers and bind to consensus DNA sequences at promoter regions of responsive genes, resulting in regulating cell proliferation, apoptosis, and differentiation [37]. Phycocyanin was reported to participate in the regulation of multiple biological processes through NF-κB signaling. For example, Li et al. had discovered that the TLR2-MyD88-NF-κB pathway played an important role in phycocyanin-mediated reduction in bleomycin-induced pulmonary fibrosis [38]. Particularly, the activity of NF-κB was greatly reduced by phycocyanin, which is in accord with our results (Figure 6B). Besides pulmonary diseases, Zhu et al. also found that phycocyanin could reduce inflammation in dextran sulfate sodium-induced colitis through inhibiting NF-κB activation in mice models [11]. Coincidentally, our previous work had confirmed their results by using a lipopolysaccharide-induced RAW 264.7 macrophage model [39]. As a matter of fact, the role of NF-κB in human cancer initiation, development, metastasis, and resistance to treatment had drawn particular attention in the past three decades [40]. It has been proven that phycocyanin could exert antitumor effects on pancreas [41] and liver [42] cancer through down-regulating NF-κB activities. Moreover, Bingula et al. detected a reduced expression of NF-κB in phycocyanin-treated A549 cells, which was also discovered in our previous work [43]. Overall, recent research had demonstrated that phycocyanin could act as a NF-κB suppressor in different biological processes, including in NSCLC A549 cells. Nevertheless, our present study suggested a regulatory mechanism of phycocyanin in multiple NSCLC cell lines, which undoubtedly provided an important theoretical basis on exploring the regulating mechanism of phycocyanin in NSCLC.

TIRAP is an important adaptor protein which belongs to the TLR/IL-1R superfamily, possessing a TIR domain in the cytoplasmic tail [26]. Studies have demonstrated that TIRAP was involved in various lung injury and repair processes. For instance, Li et al. reported that TLR4-TIRAP signaling was essential for ozone-induced lung damage repair responses [44]. In addition, TIRAP was also involved in defending against sepsis- [45] or bacterial-associated [46] acute lung injury. By contrast, our present results revealed that TIRAP participated in regulating proliferation and apoptosis of multiple NSCLC cells, which further confirmed the important role of TIRAP in different lung diseases. In fact, it has been reported that aberrant expression of TIRAP leads to the development of multiple tumors including lymphocytic leukemia [47], gastric cancer [48], colorectal cancer [49], and so on. Strikingly, Martínez-Montemayor et al. discovered that TIRAP played a key role in modulating metastatic progression of lung cancer [50], which partly supported our results. However, there has been no reports of the regulation relationship between phycocyanin and TIRAP in NSCLC. In the present work, TIRAP was identified as an important factor that took part in the phycocyanin-mediated proliferation inhibition process in H1975, H1650, and LTEP-a2 cell lines. To the best of our knowledge, this was the first investigation on the correlation of phycocyanin and TIRAP in NSCLC cells, which undoubtedly laid a theoretical basis on the potential treatment of NSCLC and the application of marine functional foods.

## Supplementary Materials

The following are available online at https://www.mdpi.com/2073-4409/8/6/588/s1, Figure S1: Validation of expressions of NF-κB pathway-related genes in LTEP-a2 cells after phycocyanin treatment, Figure S2: Western blot detection of total amounts of NF-κB signaling proteins and analysis of phosphor/total protein expression ratios.
