# Supplementary material for: Phycocyanin Exerts Anti-Proliferative Effects through Down-Regulating TIRAP/NF-κB Activity in Human Non-Small Cell Lung Cancer Cells
Source: Cells. 2019 Jun 14;8(6):588. doi: 10.3390/cells8060588 (PMC6627414; doi:10.3390/cells8060588)
Supplement: Supplementary file 1 [file cells-08-00588-s001.pdf]

Article

Phycocyanin Exerts Anti-Proliferative Effects through Down-Regulating TIRAP/NF-κB Activity in Human Non-Small Cell Lung Cancer Cells

Shuai Hao [1], Shuang Li [1], Jing Wang [1], Yan Yan [1], Xin Ai [1], Jiawen Zhang [1], Yuqing Ren [1], Tingting Wu [1], Liyun Liu [2,*] and Chengtao Wang [1,*]

1 Beijing Advanced Innovation Center for Food Nutrition and Human Health, Beijing Engineering and Technology Research Center of Food Additives, Beijing Technology and Business University, Beijing 100048, China; haoshuai@btbu.edu.cn (S.H.); lishuangldw@163.com (S.L.); trotwj960@163.com (J.W.); 15128470659@163.com (Y.Y.); 18087740320@163.com (X.A.); zhangjiawen98@outlook.com (J.Z.); RYQ512@163.com (Y.R.); m18810529269@163.com (T.W.)

2 State Key Laboratory of Infection Disease Prevention and Control, National Institute for Communicable Disease Control and Prevention, Collaborative Innovation Center for Diagnosis and Treatment of Infectious Disease, Chinese Center for Disease Control and Prevention, Beijing 102206, China.

* Corresponding authors: liuliyun@icdc.cn (L.L.); ctwangbtbu@163.com (C.W.)

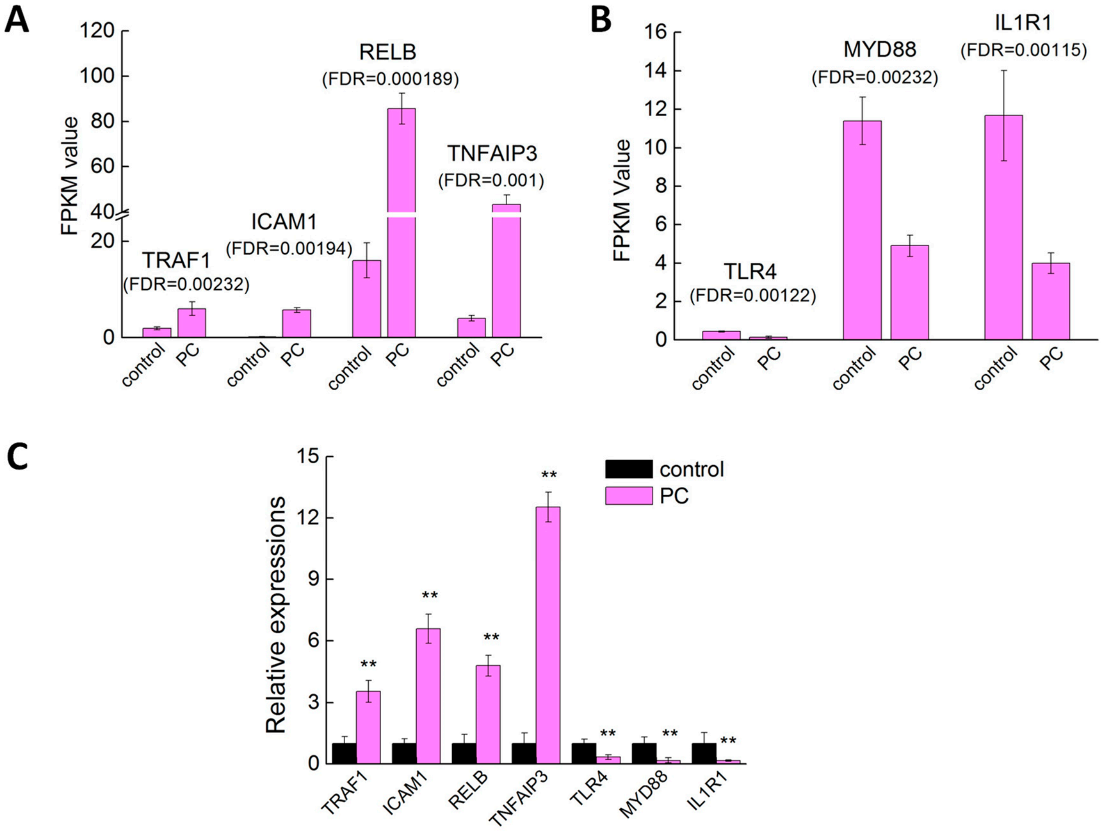

Figure S1. Validation of expressions of NF-κB pathway-related genes in LTEP-a2 cells after phycocyanin treatment. (**A**) RNA-seq analysis of TRAF1, ICAM1, RELB and TNFAIP3 expressions (up-regulated) in control and phycocyanin-treated LTEP-a2 cells. (**B**) RNA-seq analysis of TLR4, MYD88 and IL1R1 expressions (down-regulated) in control and phycocyanin-treated LTEP-a2 cells. The FDR values were marked with each detected gene. (**C**) qRT-PCR analysis of the expressions of these 7 genes in LTEP-a2 cells after phycocyanin treatment. PC, phycocyanin treatment groups. FDR, False discovery rate. Bars represent mean ± SD. **, $p < 0.01$.

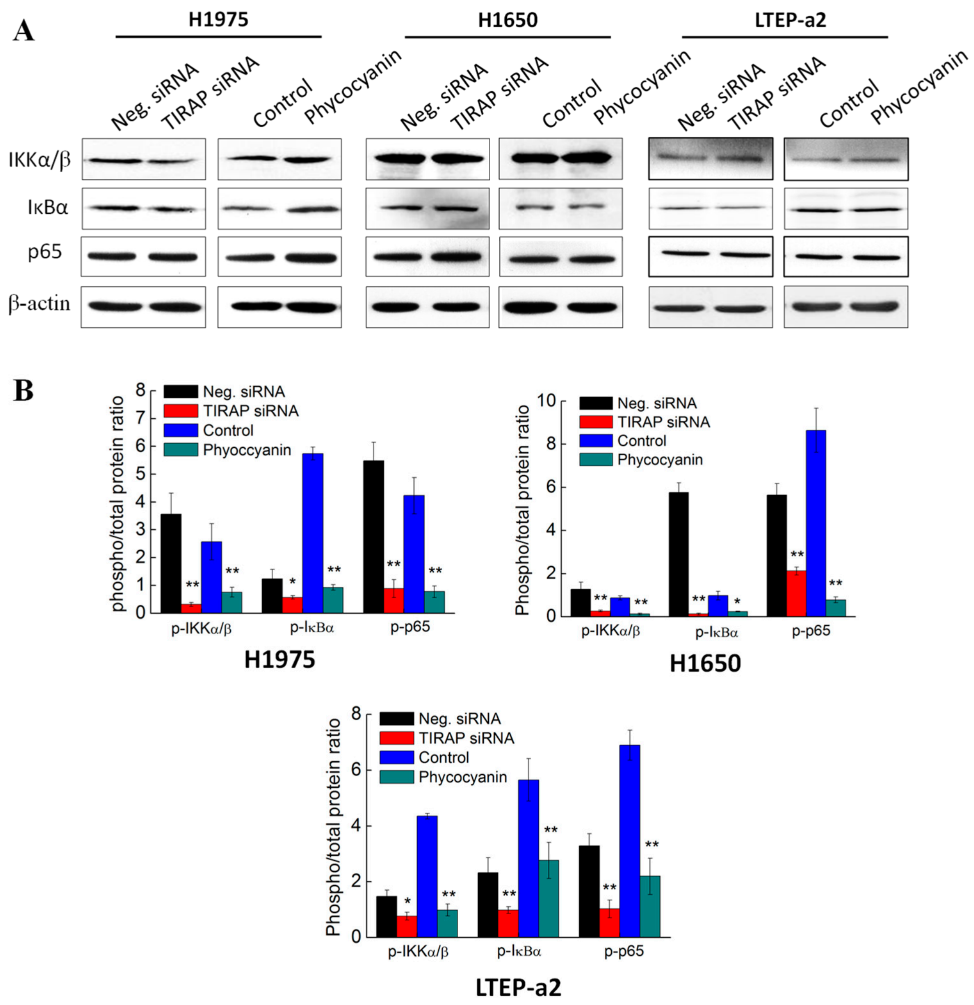

Figure S2. Western blot detection of total amounts of NF-κB signaling proteins and analysis of phosphor/total protein expression ratios. (**A**) Western blot analysis of total amounts of IKKα/β, IκBα, and p65 protein expressions in H1975, H1650 and LTEP-a2 cells after treatment with phycocyanin and TIRAP siRNA, respectively. (**B**) Phospho/total ratios of IKKα/β, IκBα, and p65 expressions in H1975, H1650 and LTEP-a2 cells after treatment with phycocyanin and TIRAP siRNA, respectively. For phycocyanin treatment, proteins were extracted after treated with phycocyanin and control for 72 h. For siRNA transfection, cells were exposed to Neg. and TIRAP siRNA for 12 h, followed by culturing with complete medium for 48 h before protein extraction. Bars represent mean ± SD. *, $p < 0.05$; **, $p < 0.01$.
